# Hybrid aortic arch procedure in acute type A aortic dissection with right carotid artery occlusion

**DOI:** 10.1093/icvts/ivad043

**Published:** 2023-03-23

**Authors:** He-Qing Wang, Ming-Kui Gao, Tie-Yan Li, Yuan-Feng Xin

**Affiliations:** Department of Cardiovascular Surgery, Shanghai East Hospital, Tongji University School of Medicine, Shanghai, China; Department of Cardiovascular Surgery, Shanghai East Hospital, Tongji University School of Medicine, Shanghai, China; Department of Cardiovascular Surgery, Shanghai East Hospital, Tongji University School of Medicine, Shanghai, China; Department of Cardiovascular Surgery, Shanghai East Hospital, Tongji University School of Medicine, Shanghai, China

**Keywords:** Aortic dissection, Carotid artery occlusion, Hybrid surgery

## Abstract

Acute type A aortic dissection complicated by carotid artery is associated with a high risk of perioperative stroke. We reported a case of application of hybrid aortic arch debranching procedure in acute type A aortic dissection complicated by right carotid artery occlusion, which resulted in no neurological complications after operation and patent carotid artery after discharging.

A 48-year-old man was hospitalized for severe back pain lasting for about 6 h. He had a history of transient blurred vision half an hour ago. Computed tomography (CT) of head showed no abnormal findings. CT angiography of aorta showed acute type A aortic dissection (ATAAD) with occlusion of right carotid artery (Fig. 1A and C). Transthoracic echocardiography showed mild–moderate regurgitation of aortic valve with normal cardiac function.

**Figure 1:**
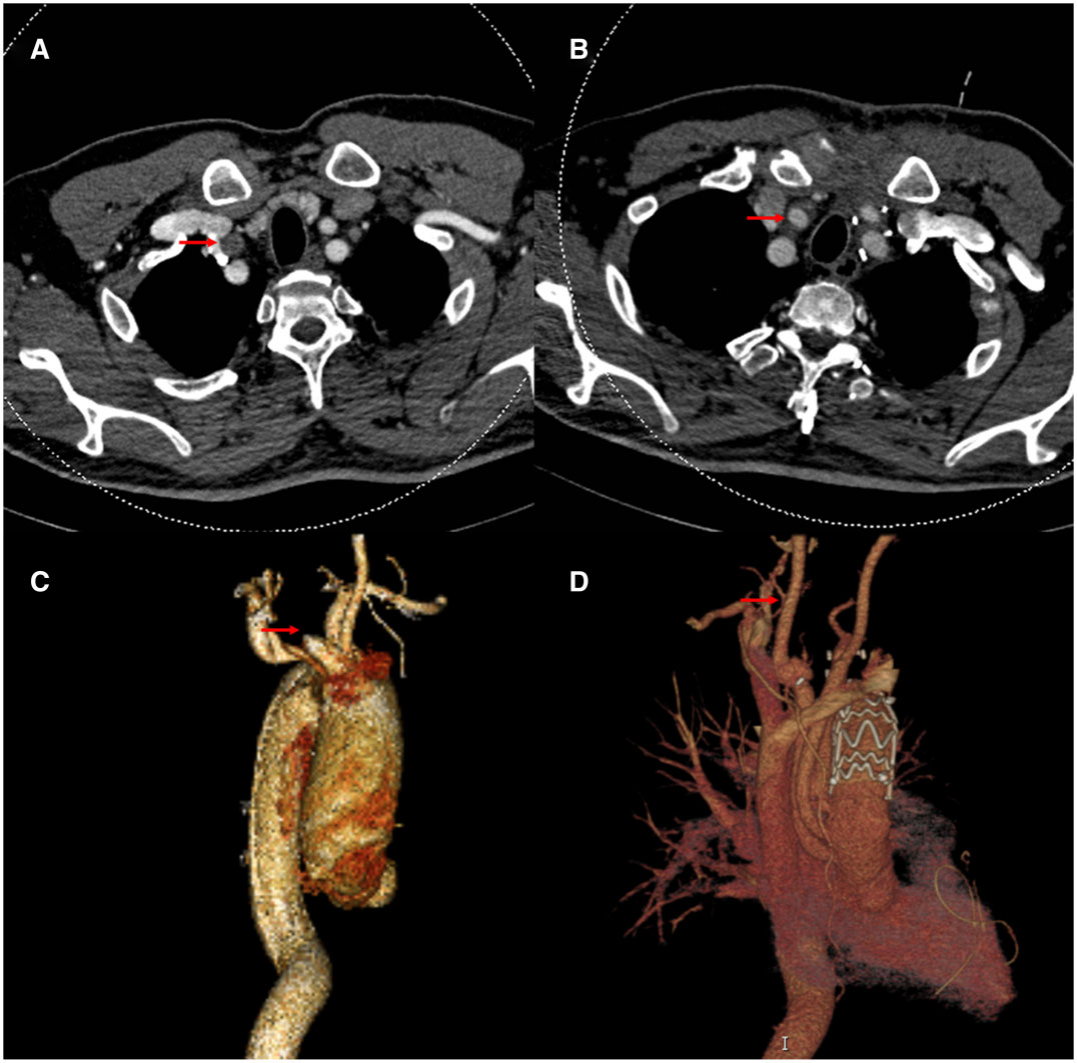
Computed tomography angiography (CTA) shows acute type A aortic dissection complicated with right carotid artery occlusion (**A** and **C**). CTA shows patent right carotid artery, successful replacement of ascending aorta and implantation of thoracic stent after discharging 3 months later (**B** and **D**).

Type II hybrid aortic arch procedure involving ascending aorta replacement, total aortic arch debranching, and thoracic endovascular stent graft implantation was implemented in our case. Cardiopulmonary bypass was established using right femoral artery perfusion, right atrium, and pulmonary vein drainage. Furthermore, brachiocephalic trunk artery was transected and trimmed to expose true lumen for selective antegrade cerebral perfusion with a balloon-tipped cannula. Systemic cooling was initiated aiming at a bladder temperature of 30°C. After cross-clamping aorta, myocardial protection was achieved using antegrade perfusion via coronary ostia with Del-Nido cardioplegia. Aortic sinus plasty was first performed with the resuspension of valve commissures and subsequent replacement of ascending aorta with 4 branches Dacron graft was implemented. Afterwards, rewarming was started and aorta was de-aired and unclamped to restore myocardial perfusion. Next, branch grafts were consecutively anastomosed to brachiocephalic trunk artery, left subclavian artery and left carotid artery. Cardiopulmonary bypass was stopped when the temperature reached 35–36°C and cannulas were removed. Finally, a thoracic endovascular stent graft was deployed landing at the graft of the last branch via right femoral artery under fluoroscopic guidance.

The patient was assisted with a ventilator for 4 days and stayed in ICU for 7 days, remaining with no neurological complications. Three months later after discharging, CT angiography of aorta showed patent right carotid artery (Fig. 1B and D) and echocardiography showed mild regurgitation of aortic valve.

## DISCUSSION

When carotid artery is involved in ATAAD, it is disputed whether repairing aorta or branch vessel first in clinical practice. In most cases, preferred repair of aorta can restore sufficient branch blood flow. It is reported that neurological injury improved in 43% after immediate aortic repair with hospital mortality of 7.0% [1]. On the contrary, branch priority may be suitable to patients who have suffered from ischaemic stroke or even conscious disturbance before operation, which rapid carotid artery stenting can improve neurological symptoms and create favourable conditions for subsequent aortic repair [2]. In our case, the patient was present with transient blurred vision solely in terms of neurological symptoms despite right carotid artery occlusion was identified in CT angiography. Therefore, emergent aortic repair surgery was adopted to restore true lumen perfusion as soon as possible.

At present, bilateral antegrade cerebral perfusion is considered a better approach to ensure sufficient cerebral blood flow under physiological condition [3]. In consideration of right carotid artery occlusion in our patient, bilateral antegrade cerebral perfusion was adopted via cannulation in right brachiocephalic trunk and femoral artery to ensure cerebral blood flow. In addition, brachiocephalic cannulation directly after cardiopulmonary bypass would reduce the ischaemic time of right carotid artery, compared with right axillary artery cannulation, resulting in less neurological complications after surgery.

Currently, hybrid arch procedure is an attractive alternative, which avoids stopping circulation and reduces operating time associated with lower rate of mortality and risk of stroke compared with conventional total arch replacement in patients over 75 years of age [4]. Our centre has applied hybrid approaches to high-risk patients with ATAAD for 5 years which achieved satisfied prognosis. In this case, confronted with carotid artery occlusion with a high risk of postoperative stroke, hybrid procedure can take full advantage of cerebral protection, resulting in no neurological complications after operation. Furthermore, distal thoracic stent implantation after proximal aortic replacement could reduce the possibility of secondary surgery by covering distal intimal tears and promoting thrombosis of false lumen, contributing to better prognosis of young type A patients, despite the risk of neurological deficit at the time of surgery when blood pressure and oxygen supply are not in optimal range.

The treatment strategy of high-risk patients with ATAAD complicated by occlusion of carotid artery varies in recent years. Hybrid one-stage aortic arch procedure may be a valid choice for patients with greater likelihood of stroke.
